# A Micro CT Study in Patients with Breast Microcalcifications Using a Mathematical Algorithm to Assess 3D Structure

**DOI:** 10.1371/journal.pone.0169349

**Published:** 2017-01-20

**Authors:** David Kenkel, Zsuzsanna Varga, Heike Heuer, Konstantin J. Dedes, Nicole Berger, Lukas Filli, Andreas Boss

**Affiliations:** 1 Department of Diagnostic and Interventional Radiology, University Hospital Zurich, University of Zurich, Zurich, Switzerland; 2 Department of Pathology and Molecular Pathology, University Hospital Zurich, Zurich, Switzerland; 3 Department of Gynecology, University Hospital Zurich, Zurich, Switzerland; Nanjing Normal University, CHINA

## Abstract

**Purpose:**

The aim of this study was to evaluate the relevance of the three-dimensional (3D) structure of breast microcalcifications (MC) as a predictor of malignancy using highly resolved micro-computed tomography (micro-CT) datasets of biopsy samples.

**Material and Methods:**

The study included 28 women with suspicious MC in their mammogram undergoing vacuum-assisted biopsy. Directly after the intervention, the specimens were scanned in a micro-CT with an isometric spatial resolution of 9 μm. Datasets were analysed regarding the number, volume and morphology of suspicious non-monomorphic MC (fl—fine linear, fp—fine pleomorphic, ch—coarse heterogeneous) and the structure model index (SMI). Histological evaluation was performed according to the B-classification: normal tissue or benign (group A: B1, B2), unclear malignant potential or suspicious of malignancy (group B: B3, B4) and malignant lesions (group C: B5).

**Results:**

In all groups, suspicious non-monomorphic MC were found: group A exhibited fp MC in 38.5% of samples, no fl/ch; group B: fl 14.3%, fp 28.6%, ch 14.3%; group C always had at least one type of suspicious non-monomorphic MC (fl (57.1%) or fp (57.1%)) in each sample. The different histologic groups showed a similar mean SMI (benign: 2.97 ± 0.31, malignant: 3.02 ± 0.10, unclear: 2.90 ± 0.28). Between the three groups, no significant differences were found regarding number, volume or SMI value of MC.

**Conclusion:**

3D structure based on the SMI of MC analysed with highest spatial resolution is not significantly associated with the B-classification of breast lesions. Thus, magnification views of MC may be omitted in the analysis of MC detected in mammograms.

## Introduction

Breast cancer is the most frequent malignancy in women, with yearly 1.38 million new breast cancer cases worldwide representing 10.9% of all new diagnosed cancer [1]. The World Health Organization (WHO) predicts that the importance of breast cancer is likely to increase in the next decades in developing and developed countries due to the increased incidence of breast cancer caused by increased life expectancy [2]. These circumstances urge an improvement in early diagnosis, firstly to improve the outcome of patients and secondly to reduce the healthcare costs related to breast cancer therapy. In Canada, for instance, the average lifetime cost per breast cancer case was higher at higher stages ($36,340 for stage IV) compared to lower stages ($23,275 for stage I) [3].

Mammography screening is the current gold standard for the early detection of breast cancer. The evaluation of mammograms is standardized according to the American College of Radiology (ACR) Breast Imaging Reporting and Data System (BIRADS) classification. One of the key findings for the detection of suspicious breast lesions and an important sign of malignancy is microcalcifications (MC) [4, 5]. Typically, the shape of the MC in breast lesions is used to classify the finding as benign or malignant [6–9]. According to the BIRADS classification, there are three categories of MC including typically benign, MC of intermediate concern and calcification with a high probability of malignancy. Benign calcifications are mostly larger, calcifications of intermediate concern are coarse heterogeneous and typical malignant MC are fine pleomorphic and fine linear [10].

Mammography is the only screening method that has been proved to be efficient and cost-effective [11–13]. The early diagnosis of breast cancer causes a decrease in the mortality associated with the disease [14]. A meta-analysis including 11 randomized trials showed that the relative risk reduction of women taking part in the screening compared to a control group not undergoing mammography screening is 20% (95% CI 0.73–0.89) [15].

The aim of this study is to evaluate whether mathematical modelling of the three-dimensional (3D) structure of MC may serve as an objective predictor of malignancy as proved by minimal invasive breast biopsy histological diagnostics.

## Methods

### Subjects

Patients with MC classified as BIRADS 2–5 without corresponding findings in the ultrasound examinations were prospectively included in this study. Twenty-nine biopsy specimens of 28 women (age range 50–86 years, mean age 60.5 ± 8.8 years) were evaluated. Informed consent was obtained from each patient; all patients signed a written informed consent that their tissue can be used for further imaging and histological research. The Department of Pathology and Molecular Pathology, University Hospital Zurich included this study into a larger breast pathology project (KEK-2012-554). There was no need for a specific approval of this subproject by the local ethical committee, as informed consent was available in each patient and only the histological diagnosis of the minimal invasive breast biopsies were included in the study without any further analyses on the breast tissues.

### Mammography

The patients were examined using a mammography system (MAMMOMAT Inspiration; Siemens AG, Healthcare Sector, Erlangen, Germany) in the two standard projections: mediolateral oblique view and craniocaudal view. All mammographies were classified according to the BIRADS classification [7]. Prior to biopsy, mammographies were discussed by an interdisciplinary board including radiologists and gynaecologists; after biopsy, histological results were discussed by an interdisciplinary board between pathologists, radiologists and gynaecologists.

### Breast biopsy

Patients underwent vacuum-assisted stereotactic breast biopsy using an EnCor Enspire breast biopsy system (Bard, Tempe) with a needle size of 7 gauge or 10 gauge. Patients were rested in a prone position on the biopsy table. The biopsies were executed by a senior attending physician from the Department of Gynaecology or the Department of Radiology. Immediately after extraction, samples were fixed in formalin.

### Micro-CT imaging

Each specimen was removed from the formalin-filled tube and covered with plastic wrap in order to protect the sample from drying during measurement. Imaging was performed on a micro-CT system (SkyScan 1176, Bruker microCT, Kontich, Belgium). The biopsies were positioned in the isocentre of the micro-CT bed and stabilized with adhesive tape. Images were acquired after a localizer scan using the following scan parameters: rotation step 0.5°, tube voltage 90 kV, tube current 280 μA, covered angle 360°, copper filter 0.1 mm, voxel size 9 × 9 × 9 μm^3^. The total scanning time for each sample was 120 min. Images were then reconstructed using a modified Feldkamp cone-beam algorithm (NRecon, SkyScan/Bruker microCT, Kontich, Belgium). The applied MicroCT Skyscan 1176 uses a Feldkamp filtered backprojection reconstruction for cone-beam CTs. In principle, the reconstruction of each single image is performed in two dimensions, however, as a volume dataset is acquired the 2D slice are reconstructed with isotropic voxel size.

### Image analysis

After reconstruction, different morphological parameters were calculated using CTAn/CTVol (SkyScan/Bruker microCT) including the object volume and SMI. For assessment of the 3D structure of the MC the structure model index (SMI) was calculated. SMI is a dimensionless quantity, that is defined according to the following equation of Hildebrand et al [16]:

$$SMI\;=\;6\;\times \;\left(\frac{\text{S}’\times \text{V}}{S^{2}}\right)$$

where S is the object surface area before dilation, S’ is the change in surface area caused by dilation and V is the undilated object volume. SMI ranges from 0 to 4: a value of 0 signifying a plate-like structure, 3 a cylindrical structure and 4 a sphere. Intermediate values indicate a mixed structure. Further, for each sample the mean object volume and the diameter assuming spherical and cubic shape were calculated.

In analogy to the BIRADS classification, the MC were categorized according to the following scheme: fine linear (fl) MC defined as calcifications with a mean estimated diameter (cube) of < 200 μm and an SMI of 2.92–3.08, fine pleomorphic (fp) with a mean estimated diameter (cube) of < 250 μm and a standard deviation (SD) of the SMI of more than 0.29, coarse heterogeneous (ch) defined as calcifications with an estimated diameter > 500 μm and an SD of the SMI > 0.5 and no suspicious MC defined if none of the other definitions is accurate.

### Histological evaluation

The specimens were fixed in 4% formaldehyde and processed and stained according to the standard procedures including serial sections and hematoxylin and eosin staining. Histological diagnoses were made based on the current B-classification [17]. The B1 category indicates normal tissue that might show MC, e.g. within involutional lobules. The B2 category includes benign lesions such as fibroadenomas, fibrocystic changes, ductectasia and sclerosing adenosis. B3 lesions are of uncertain malignant potential including atypical ductal hyperplasia (ADH), classical lobular neoplasia (LN), phylloides tumours and papillary lesions. The B4 category contains suspicious lesions for malignancy, but not affirmative diagnostic for malignancy. Lesions categorized as B5 lesions are definitely malignant [18, 19].

### Statistical analysis

The mean and SD of the size and the morphological parameters were calculated. Data were tested for normal distribution using the Kolmogorov–Smirnov (KS-test). The means of the morphological parameters of the different histological groups were compared using an independent samples Kruskal–Wallis test and the chi-square test was used to compare the amount of BIRADS-MC with the histological groups, including those which were histologically proven to be normal or benign (group A: B1 and B2), of uncertain potential or suspicious (group B: B3 and B4) and malignant (group C: B5). Due to the multiple comparisons, a Bonferroni correction was performed. The data were analysed using IBM SPSS-Statistics version 22. A p-value of less than 0.05 was considered to be significant.

## Results

### Subjects and histology

In 29 biopsies from 28 patients 829 MC were detected resulting in a mean number of MC of 28.6 ± 34.7 per specimen. The majority of the examined patients were radiologically considered to be BIRADS 4 (n = 25), two were assessed as BIRADS 5, one lesion was classified as BIRADS 2 and one lesion as BIRADS 3. The histology showed that 8 of 27 biopsies were malignant (29.6%). Table 1 gives an overview of the patients age, the histological (B-classification) and radiological (BIRADS) evaluation. For the morphologic evaluation, two samples containing just one MC were excluded. At the interdisciplinary board these biopsies were considered to be non-representative since more than one MC was observed on the mammograms.

**Table 1:** Patient age and overview of the histologic (B-classification) and radiologic (BIRADS) evaluation.

Sample number	B-classification	BIRADS	A, normal or benign B, unclear biological potential or suspicious C, malignant	Patient age (years)	Histological diagnosis
1	B2	4	A	52	Fibrosis, apocrine metaplasia
2	B1	4	A	58	Fibrosis, no calcifications
3	B2	4	A	58	Fibrosis with calcifications
4	B2	4	A	63	Fibrosis, sclerosing adenosis
5	B2	4	A	57	Fibrosis, sclerosing adenosis
6	B2	4	A	65	Fibrosis, sclerosing adenosis, usual ductal hyperplasia, intraductal papilloma.
7	B2	4	A	57	Fibroadenoma
8	B2	4	A	52	Fibrosis, sclerosing adenosis
9	B1	2	A	60	Lipomatosis and ectasia of the ducts
10	B2	4	A	53	Usual ductal hyperplasia
11	B2	4	A	74	Sclerosing adenosis, usual ductal hyperplasia,
12	B2	4	A	59	Fibrosis, usual ductal hyperplasia
13	B2	4	A	54	Fibrotic duct
14	B2	4	A	54	Lipomatosis
15	B4	4	B	60	ADH
16	B3	4	B	65	Intraductal papilloma, apocrine metaplasia, usual ductal hyperplasia
17	B3	4	B	54	ADH in an inflammatory lobulus
18	B3	4	B	52	Flat epithelial atypia
19	B3	4	B	52	Sclerosing adenosis, fibrosis, microcalcifications at the end of the ducts
20	B3	4	B	86	Classical Lobular neoplasia, sclerosing adenosis, scar
21	B3	4	B	52	Classical Lobular neoplasia
22	B5	5	C	76	DCIS high grade
23	B5	4	C	52	DCIS non-high grade
24	B5	5	C	49	DCIS high-grade and lymphangiosis carcinomatosa
25	B5	4	C	60	DCIS high grade
26	B5	4	C	69	Invasive carcinoma (ductal type,NST)
27	B5	4	C	63	DCIS high-grade in transition to invasive carcinoma (ductal type, NST)
28	B5	4	C	70	DCIS mainly non high grade focally high grade
29	B5	3	C	74	DCIS non high grade

### Morphological evaluation using micro-CT

**Fig 1:**
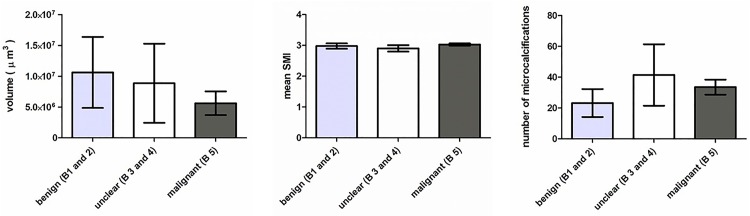
Overview of different morphological parameters including mean number, volume (μm^3^) and SMI of MC for all different groups (benign: including B-classification B1 and B2; unclear malignant potential: including B-classification B3 and B4; malignant lesions: including B-classification B5). Error bars indicate the standard error of the mean (SEM).

Table 2 reports the results of the morphological parameters (number of MC, mean object volume, morphology, mean SMI) for each sample. No significant differences between benign (group A) lesions, lesions with unclear malignant potential (group B) and malignant (group C) lesions were observed regarding the number of MC (P = 0.113), object volume (P = 0.881), mean SMI (P = 0.756) or the SD of the SMI (P = 0.976). Table 3 and Fig 1 show the results of the different morphological parameters including number of MC, object volume and mean SMI for group A–C lesions. The different histologic groups exhibited a similar mean SMI (group A: 2.97 ± 0.31, group B: 2.90 ± 0.28, group C: 3.02 ± 0.1) and SD of the SMI (group A: 0.32 ± 0.19, group B: 0.32 ± 0.18, group C: 0.31 ± 0.11. Moreover, group C lesions (33.6 ± 12.8) showed a tendency to comprise fewer calcifications than group B lesions (41.4 ± 52.7) and more than group A lesions (23.2 ± 32.6), without reaching significance.

**Table 2:** Morphological parameters including object volume (μm^3^x10^5^) and the structure model index (±SD) of every specimen. Morphology: fl: fine linear, fp: fine pleomorphic, ch: coarse heterogeneous, ns: no suspicious MC, 1 indicating presence and 0 indicating absence of the respective type of microcalcification.

Sample Number	Group	Number of MC	Mean object volume [μm^3^x10^5^]	Morphology				SMI mean (±SD)
				fl	fp	ch	ns	
1	A	23	16	0	1	0	0	3.27±0.33
2	A	1	1	1	0	0	0	3.02±0
3	A	13	65	0	1	0	0	2.95±0.39
4	A	5	6	0	1	0	0	2.79±0.45
5	A	11	30	0	0	0	1	3.14±0.18
6	A	53	28	0	0	0	1	3.30±0.28
7	A	18	325	0	0	0	1	2.76±0.35
8	A	121	43	0	0	0	1	3.29±0.29
9	A	10	82	0	0	0	1	3.07±0.12
10	A	5	25	0	1	0	0	2.19±0.80
11	A	31	13	0	0	0	1	3.30±0.18
12	A	2	2	0	0	0	1	2.86±0.04
13	A	6	737	0	0	0	1	2.89±0.24
14	A	4	7	0	1	0	0	2.89±0.45
15	B	5	473	0	0	1	0	2.59±0.60
16	B	36	40	0	1	0	0	2.52±0.45
17	B	7	9	0	0	0	1	3.14±0.09
18	B	150	22	0	0	0	1	3.15±0.24
19	B	15	37	0	1	0	0	2.75±0.46
20	B	67	22	1	0	0	0	3.07±0.24
21	B	10	16	0	0	0	1	3.10±0.20
22	C	58	36	1	0	0	0	2.95±0.25
23	C	25	14	1	0	0	0	3.00±0.27
24	C	30	15	0	1	0	0	2.87±0.54
25	C	31	19	1	0	0	0	3.03±0.20
26	C	1	3	0	0	0	1	2.79±0
27	C	30	87	0	1	0	0	3.11±0.31
28	C	19	152	0	1	0	0	3.15±0.30
29	C	42	70	1	1	0	0	3.08±0.30

**Table 3:** Overview of the results of different morphological parameters including mean SMI, SD SMI, mean number of calcifications, the object volume for histologically proven benign, malignant and unclear biological potential lesions. Moreover the presence of the morphological MC types in the different groups is reported.

	Group A: benign	Group B: unclear malignant potential	Group C: malignant
**Morphological parameters**			
Mean SMI	2.97±0.31	2.90±0.28	3.03±0.1
SD SMI	0.32±0.19	0.32±0.18	0.31±0.11
Object volume (μm^3^x10^5^)	106±207	88±169	56±50
Mean number of microcalcifications	23.2±32.6	41.4±52.7	33.6±12.8
**Morphology of MC**			
Fine linear	0/13	1/7	4/7
Fine pleomorphic	5/13	2/7	4/7
Coarse heterogeneous	0/13	1/7	0/7
No suspicious calcification	8/13	3/7	0/7

### Categorization of MC

Table 2 reports the presence of different types of MC for each specimen and Table 3 provides an overview for group A–C lesions. Group A lesions showed no fine linear or coarse heterogeneous MC; fine pleomorphic were found in 38.5% (5/13) of the samples and no suspicious MC were found in 61.5% (8/13). Group B lesions exhibited a more heterogeneous distribution of MC types than group A and C lesions: fine linear in 14.3% of samples (1/7), fine pleomorphic in 28.6% (2/7), coarse heterogeneous in 14.3% (1/7) and no suspicious calcification in 42.9% (3/7) of the samples. Group C lesions always showed at least one type of suspicious MC: 57.1% of the samples exhibited fine linear (4/7) and 57.1% showed fine pleomorphic MC (4/7).

A chi-square test demonstrated that the absence of suspicious MC between groups A–C was not significant after Bonferroni correction (P = 0.028); however, the presence of fine linear MC was significantly different between groups A–C (P = 0.007). No significant differences were observed regarding the presence of fine pleomorphic (P = 0.539) and coarse heterogeneous MC (P = 0.227) between the groups.

**Fig 2:**
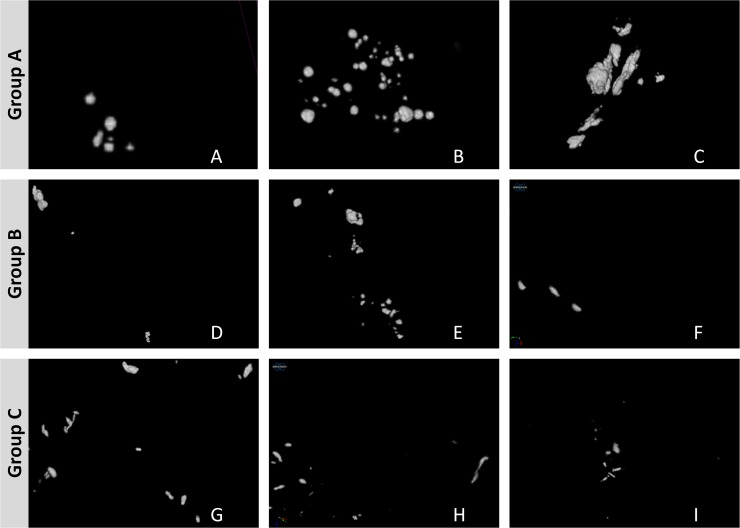
Representative 3D images from different biopsies. Benign (group A: A–C) lesions, unclear biological potential lesions (group B: D–F) and malignant (group C: G–I) lesions: A (sample number 1, fine pleomorphic), B (sample number 6, no suspicious MC), C (sample number 7, no suspicious MC), D (sample number 15, coarse heterogeneous), E (sample number 16, fine pleomorphic), F (sample number 20, fine linear), G (sample number 22, fine linear), H (sample number 25, fine linear) and I (sample number 24, fine pleomorphic). For more details concerning the different samples, compare sample numbers in Tables 1 and 2.

**Fig 3:**
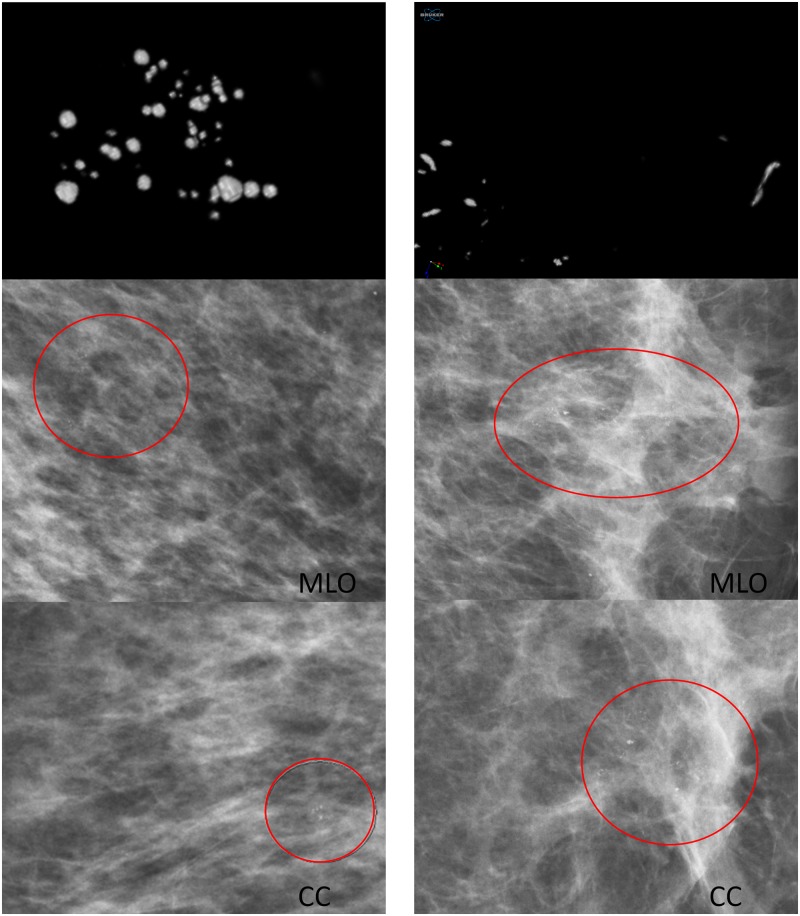
Representative 3D images and mammograms from two different patients. Left column: sample number 6 (fibrosis, sclerosing adenosis, normal ductal hyperplasia, intraductal papilloma), only spherical MC classified as no suspicious MC; right column: sample number 25 (DCIS high grade), fine linear MC. CC indicates craniocranialcaudal view and MLO indicates mediolateral oblique view.

Fig 2 shows representative 3D images from different biopsies from group A (A–C), group B (D–F) and group C lesions (G–I). Typically, group C lesions (H and I) exhibited fine linear MC more often than group A lesions, which never showed a fine linear pattern (A–C). Group B lesions showed the broadest variety of MC including all kinds of MC and also lesions with no suspicious MC. In Fig 3, it is demonstrated that the different types of MC can easily be assessed in micro-CT images due to the excellent spatial resolution.

## Discussion

In the present study, we examined the form and morphological characteristics of MC detected by mammography. Samples from vacuum-assisted stereotactic breast biopsy underwent micro-CT measurements with a highest spatial resolution of 9 μm; the MC were 3D-segmented with subsequent morphological analysis using an automatic algorithm providing the number of MC, volume and SMI. Moreover, MC were categorized as suspicious MC exhibiting a fine linear, fine pleomorphic or coarse heterogeneous pattern. None of the morphological parameters or any of the types of suspicious MC showed a statistical correlation to the B-classification type of the lesion. Therefore, the 3D shape of the MC does not allow benign lesions to be distinguished from lesions with unknown malignant potential or breast cancers.

In our study, 93% of the examined patients showed lesions radiologically classified as BIRADS 4 and 5; in the histological examination of the biopsy samples 29.6% were classified as B5 lesions corresponding to a positive predictive value (PPV) of suspicious MC in mammograms of approximately 30% at our institution. This estimated PPV is in accordance with other studies reporting a PPV in the order of 30–50% for MC undergoing biopsy [4, 5]; therefore, it may be assumed that a typical mammography screening cohort was evaluated in this prospective analysis.

Only one other study assessing the 3D shape of MC using micro-CT has been previously published, by Willekens et al [20]. Compared to the Willekens study, we applied a substantially higher isotropic spatial resolution of 9 μm compared to 35 μm. Further, Willekens et al excluded MC that were smaller than three micro-CT voxels, only analysing calcifications larger than 105 μm. Furthermore, a nearly three times larger sample size was included in our study, compared to the 11 samples evaluated in the Willekens study. Some of our findings are in line with the data of Willekens et al: Patients with histologically proven benign lesions had fewer MC than those with malignant lesions; this finding was, however, not significant in our study. Likewise, our study demonstrated that SMI values do not allow distinction between malignant and benign lesions.

The category of B3 lesions comprises a heterogeneous group of different histological lesion types including atypical hyperplasia (AH) with ADH and classical LN including atypical lobular hyperplasia (ALH) and classical lobular carcinoma in situ, flat epithelial atypia (FEA), phylloides tumour, papillary lesions and radial scars. Often, B3 lesions undergoing resection are later upgraded to B5 lesions because of a small invasive focus in the removed tissue [19, 21]. The B3 and B4 lesions included in our study showed a wide variety of different types of MC with one type of suspicious MC. However, two preliminary aspects were discovered for which a high resolved depiction of MC might provide additional helpful information. Statistically significant differences between groups A and C were detected regarding the presence of fine linear MC. Fine linear MC were only present in groups B and C; therefore, the subtype of fine linear MC seems to be a potential predictor of malignancy or lesions of unknown malignant potential. Moreover, each malignant lesion showed at least one type of suspicious MC, whereas benign lesions in the majority of cases did not show suspicious MC, demonstrating that the absence of suspicious MC might be a weak predictor for the benignancy of a lesion. In our study these differences were not significant; this could be attributed to a sample size that was too low to detect such rather weak correlations.

Several approaches have been undertaken to improve the assessment of MC including X-ray magnification views [4, 6, 22–29] and computer-aided detection with partially successful results: the contrast-limited adaptive histogram equalization (CLAHE) technique for example showed promising results for the computer-aided detection in digital mammograms with a sensitivity of 92.54% and a specificity of 92.50% [30]. However, assessment of the real 3D structure of MC remains challenging in 2D projection views. A technique offering the possibility of obtaining a 3D view is breast tomosynthesis [31]. If tomosynthesis could reach a resolution high enough to distinguish fine linear MC from fine pleomorphic MC, an increase of the PPV of X-ray breast imaging with a reduction of over-diagnosis and avoidance of unnecessary biopsies might become feasible. Although tomosynthesis might be a promising tool in the future it has to be taken into account that, in general, CT images suffer from noise and artifacts, especially in low dose mode. Therefore, previously published special low dose imaging algorithms should be implemented [32–36].

Our study also has limitations: (a) a relatively small sample size was evaluated with heterogeneous findings regarding histology. A larger cohort might provide a deeper insight into whether the fine linear MC subtype exhibits a predictive value for the presence of malignancy. It would be desirable to have a database like the Mammographic Image Analysis Society (MIAS) database of digital mammograms. This database contains 322 digitizeddigitised films. To the best of our knowledge there is no public micro-CT database of breast MC. However, we believe that a database would be desirable for the assessment of MC since it would allow the evaluation of a higher sample size. (b) Further, we only assessed the micro-CT images alone. Consecutively, other predictors of malignancy like the distribution of calcification, which is just visible on the mammograms, were not taken into account. (c) We only assessed the Structural Model Index SMI. However, it has to be mentioned that there are many other descriptors for the 3D structure, e.g. fractal dimension [37]. However, the evaluation of other assessment measures was beyond the scope of this study.

In conclusion, we showed that the shape (based on the SMI) of MC assessed even with highest spatial resolution using micro-CT is not significantly associated with the B-classification of the underlying lesion, especially regarding the presence of malignancy. Taken together, it seems to be justified to omit magnification views for the assessment of MC.
